# The Accelerated Progression of Atherosclerosis Correlates with Decreased miR-33a and miR-21 and Increased miR-122 and miR-3064-5p in Circulation and the Liver of ApoE-/- Mice with Streptozocin (STZ)-Induced Type 2 Diabetes

**DOI:** 10.3390/cimb44100328

**Published:** 2022-10-13

**Authors:** Hui-Yu Luo, Gan Li, Yu-Guo Liu, Yuan-Hao Wei, Jun-Bin Chen, Xiang-Fu Gu, Jia-Qi Tang, Yue Zhao, Chu-Hong Su, Ling-Yu Xiao, Fei Xiong, Zhong-Daixi Zheng, Shi-Ying Wang, Long-Ying Zha

**Affiliations:** Department of Nutrition and Food Hygiene, Guangdong Provincial Key Laboratory of Tropical Disease Research, National Medical Products Administration (NMPA) Key Laboratory for Safety Evaluation of Cosmetics, School of Public Health, Southern Medical University, Guangzhou 510515, China

**Keywords:** Atherosclerosis, type 2 diabetes (T2D), microRNA, cholesterol efflux

## Abstract

Atherosclerosis is a major risk factor for type 2 diabetes (T2D) mortality. We aim to investigate the changes in miR-21, miR-122, miR-33a and miR-3064-5p in circulation and the liver of ApoE-/- mice with streptozocin (STZ)-induced T2D. Twenty 5-week-old male ApoE-/- mice were randomly assigned to the control (*n =* 10) and T2D group (*n =* 10) and intraperitoneally injected with a citrate buffer and streptozotocin (STZ) (40 mg/kg BW) once a day for three consecutive days. The successfully STZ-induced T2D mice (*n =* 5) and control mice (*n =* 5) were then fed with a high-fat diet (HFD) for 34 weeks. Compared to the control mice, ApoE-/- mice with STZ-induced T2D had slower (*p* < 0.05) growth, increased (*p* < 0.05) total cholesterol (TC) and low-density lipoprotein cholesterol (LDL-C), decreased (*p* < 0.05) high-density lipoprotein cholesterol (HDL-C) in serum, reduced (*p* < 0.05) TC and sterol regulatory element-binding protein-2 (Srebp-2), elevated (*p* < 0.05) ATP-binding-cassette-transporter-A1 (Abca1) in the liver, aggravated (*p* < 0.05) atherosclerotic lesions in the aorta, downregulated (*p* < 0.05) miR-21 and miR-33a, and upregulated (*p* < 0.05) miR-122 and miR-3064-5p in serum and the liver. In addition, the aortic lesions showed a positive correlation with miR-122 (*r* = 1.000, *p* = 0.001) and a negative correlation with miR-21 (*r* = −1.000, *p* = 0.001) in ApoE-/- mice with T2D. In conclusion, T2D-accelerated atherosclerosis correlates with a reduction in miR-21 and miR-33a and an elevation in miR-122 and miR-3064-5p in circulation and the liver of ApoE-/- mice.

## 1. Introduction

Atherosclerosis is a widespread chronic disease and a major risk factor for myocardial infarction, stroke, and ischemic gangrene, and is a leading cause of morbidity and mortality worldwide [1]. It is caused by both genetic and environmental factors with complex pathogenesis [2]. Studies have shown that atherosclerosis and diabetes mellitus (DM) are connected. DM is a group of carbohydrate metabolism disorders and is mainly featured by chronic hyperglycemia due to the defects of insulin secretion and/or insulin action. The International Diabetes Federation (IDF) estimates that 415 million people worldwide have diabetes, 91% of whom have type 2 diabetes (T2D) [3]. The increased risk and accelerated progression of atherosclerosis have been found in diabetic patients. For instance, adolescents and children with type I diabetes mellitus (T1D) exhibit the early development of atherosclerosis [4]. In type 2 diabetes (T2D) patients, coronary atherosclerosis is often accelerated with the enlargement of the necrotic core size, more aggravated inflammatory infiltrates, and more diffuse plaques in the coronary arteries [2]. In recent years, although the incidence of cardiovascular and cerebrovascular disease in diabetic patients has been decreasing, it is still the leading cause of death and disability in patients with T2D [5]. According to recent studies, 29.1% of patients with T2D had atherosclerosis [6]. Numerous studies have investigated the common pathological connections between atherosclerosis and diabetes mellitus [2,7]. Several factors including dyslipidemia with increased levels of atherogenic low-density lipoprotein (LDL), hyperglycemia, insulin resistance, oxidative stress and inflammation are proposed to explain the acceleration of atherosclerosis in diabetes mellitus. However, the pathological factors affecting the accelerated development of atherosclerosis in diabetes are still not completely understood. Understanding the underlying mechanisms is crucial for identifying new potential molecular targets [7]. In recent years, accumulating evidence has suggested the role of microribonucleic acids (miRNAs) in mediating the metabolic transition between atherosclerosis and diabetes.

MiRNAs are endogenous, approximately 22 nucleotide-long non-coding RNAs that post-transcriptionally inhibit gene expression by altering the stability of messenger RNAs (mRNAs) and/or repressing mRNAs translation via pairing to the 3′-untranslated regions (3′-UTR) of target mRNAs of protein-coding genes [8]. MiRNAs have evolutionary importance in the modulation of gene expression since many of them are highly conserved across species. A single mRNA may be regulated by several various miRNAs, so each miRNA may have multiple mRNA targets [9]. MiRNAs are found in distinct biofluids like blood, saliva, and urine and show remarkable stability due to their packaging into vesicles [10]. The MiRNA pattern in biofluids will change under pathological conditions. The relationship between miRNAs and diseases has high complexity [11]. Thus, it is more promising to detect the combinations of multiple miRNAs (miRNA signature) rather than a single miRNA when discovering the integral role of miRNAs in diseases. MiRNAs have shown promise for use in diagnostic or prognostic tests in diseases such as atherosclerosis, T2D, etc. [12].

Atherosclerosis is mainly characterized by lipid accumulation and chronic inflammation in the arterial wall. Several miRNAs (miR-21, miR-122, miR-33a and miR-3064-5p) have been shown to be associated with lipid metabolism and inflammation, thus possibly participating in the development of atherosclerosis [9,10,11,12,13]. However, the role of these miRNAs in the development of atherosclerosis in the case of T2D is still far from being completely understood. Therefore, the objective of this study is to investigate the changing pattern of miR-21, miR-122, miR-33a and miR-3064-5p in the circulation and liver of ApoE-/- mice with streptozocin (STZ)-induced T2D and to identify their potential as biomarkers for screening cardiovascular risks in T2D.

## 2. Materials and Methods

### 2.1. Animals and Treatments

All animal care and intervention procedures in this study were approved by the Southern Medical University Animal Care and Use Committee (no. SMUA2017003).

Twenty 5-week-old male ApoE gene knockout (ApoE-/-) C57BL/6J (B6) mice (no. 312024300001195) were obtained from Shanghai Southern Model Biological Research Center (Shanghai, China). All mice were housed in standard cages in a room maintained at 22 ± 1 °C on a 12 h/12 h light/dark cycle and fed with a high-fat diet (HFD, no. D12451, Guangdong Medical Laboratory Animal Center, Guangzhou, China) containing 45% fat with an energy level of 4.73 kcal/g. Diets and sterile water were provided ad libitum.

After 1 week of adaptive feeding, all mice were randomly assigned to the control (*n =* 10) and type 2 diabetes (T2D) group (*n =* 10). Following an intraperitoneal glucose tolerance test (IPGTT), mice in the T2D group were given an intraperitoneal injection of STZ (dissolved in 0.1 M citrate buffer, pH 4.5; catalog number. S0130, Sigma, St. Louis, MO, USA) once a day at a dose of 40 mg/kg body weight (BW) for three consecutive days. Meanwhile, mice in the control group received an intraperitoneal injection of 3 mL/kg BW citrate buffer (0.1 M, pH 4.5). After the three days of injections, the random blood glucose levels of all mice were detected once a day for another three consecutive days using a glucometer. The IPGTT was performed once again.

Mice in the T2D group were considered diabetic by using the standards as follows: (1) the random blood glucose level ≥ 11.1 mmol/L, and (2) the area under the curve (AUC) of IPGTT > the average AUC of IPGTT of all mice in the control group. Based on these standards, five mice (*n =* 5) were finally judged as diabetic and the other five mice were excluded as non-diabetic. Correspondingly, only five mice (*n =* 5) from the control group were randomly selected and remained.

All mice (*n =* 5 in the control group and *n =* 5 in the T2D group) were then fed with an HFD for 34 weeks. BW and feed consumption were recorded once a week. The random blood glucose was determined once a week. At the end of the feeding experiment, the mice were fasted overnight, placed in a closed box with a concentration control valve, anesthetized with ether, and blood samples collected, and then sacrificed for the collection of the other samples.

### 2.2. Intraperitoneal Glucose Tolerance Test (IPGTT)

Three days before and after the intraperitoneal injection of STZ or citrate buffer experiment, mice were fasted overnight and intraperitoneally injected with 2 mg/g BW of glucose [14]. Blood glucose levels at 0, 15, 30, 60, 90 and 120 min after intraperitoneal glucose injection were measured using the Roche ACCU-CHEK Performa glucometer (Indianapolis, IN, USA). The blood glucose curve was plotted and the AUC was calculated using GraphPad Prism 5.0 software.

### 2.3. Oil Red O Staining for Aortic Plaque Lesions

After the heart was perfused with phosphate-buffered solution (PBS), the entire aorta was exposed under the dissecting microscope, carefully separated in its entirety, and fixed with 4% paraformaldehyde for 48–72 h. After fixation, the adipose tissues around the aorta were fully peeled off. Then the aorta was opened longitudinally with ophthalmic scissors and then fixed to a black dish in a horizontally tiled manner by steel needles. The aorta was cleaned three times with PBS to remove any floating impurities. The aorta was stained with oil red O at room temperature for one hour, then rinsed with PBS three times and photographed with a microscope with a Canon camera [15]. The Image-Pro Plus 6.0 software was used to calculate the proportion of red lipid plaques in the entire aorta.

### 2.4. Analysis of Biochemical Parameters in Serum and Liver Tissues

Following overnight fasting and anesthetization of the mice, blood was collected from the retro-orbital plexus, separated, and the serum stored at −80 °C. Commercially available enzyme-linked immunosorbent assay (ELISA) kits (ExCell Biotech, Shanghai, China) were used to determine the level of insulin in the serum. The levels of triglyceride (TG), total cholesterol (TC), high-density lipoprotein cholesterol (HDL-C) and low-density lipoprotein cholesterol (LDL-C), in the serum and liver, were determined using the corresponding commercial kits (Nanjing Jiancheng Bioengineering Institute, Nanjing, China) as per the manufacturer’s recommended protocols.

The blood glucose level without fasting was randomly determined using the glucometer once a week.

### 2.5. Real-Time Fluorescent Quantitative Polymerase Chain Reaction (PCR)

Total RNA was extracted from samples using the TRIZOL reagent (Invitrogen, Carlsbad, CA, USA) by following the manufacturer’s instructions. Aliquots of 0.5 μg RNA were reverse transcribed to cDNA using the PrimeScript RT reagent kit (AG, Guangzhou, China). A stem-loop primer was used for microRNA reverse transcription, and a universal primer in the PrimeScript RT reagent kit was used for the reverse transcription of U6. Quantitative PCR (QPCR) was performed using the LightCycler 96 (Roche, Indianapolis, IN, USA). QPCR was performed using the SYBR Green Realtime PCR Master Mix (AG, Guangzhou, China) [16], and the reaction mixtures were incubated at 95 °C for 1 min, followed by 40 cycles of 95 °C for 2 s and 60 °C for 20 s and 70 °C for 10 s. Experiments were replicated at least three times. The primers used in this study were listed in Table 1. The relative expression of miRNAs was evaluated using the 2^−ΔΔ Ct^ method and normalized to the expression of U6, respectively.

### 2.6. Western Blotting

50 mg of liver tissue was taken from each mouse and placed in a 1.5 mL EP tube. The tissue was soaked in a liquid containing 1% phenylmethylsulfonyl fluoride (PMSF) and cut into pieces of about 3 mm × 3 mm using ophthalmic scissors. PBS supernatant was removed by centrifugation after shaking the EP tube, and this step was repeated three times. Then, 400 μL cold radioimmunoprecipitation assay (RIPA, KeyGEN Biotech, Nanjing, China) containing 1% PMSF and 1% phosphatase inhibitor was added to the tube and three steel balls were added. Then, the tube was put into a low-temperature grinding machine (Xavier Biotechnology, Wuhan, China), at 70 Hz, for 60 s, and run twice. The supernatant was collected after being centrifuged at 10,000 rpm for 5 min at 4 °C, and the protein concentration was determined by the bicinchoninic acid (BCA) method.

Western blotting was performed as described earlier [17]. In simple terms, a 1.0% thick tris–glycine gel was prepared and liver tissue protein samples were added at a loading of 40 μg, and the marker was added. Electrophoresis conditions were 80–120 V. The proteins of the gel were then transferred to the nitrocellulose membrane (Bio-Rad) at a voltage of 100 V. The membrane was subsequently treated with 5% bovine serum albumin in TBST for 120 min at 25 °C to prevent non-specific reactions before incubating with an anti-Abca1 antibody (1:1000; catalog number. 96292s, Cell Signaling Technology, Danvers, MA, USA), or anti-Srebp-2 antibody (1:1000; catalog number. 28212-1-AP, Proteintech, Wuhan, China), or anti-β-actin antibody (1:5000; catalog number. 4967s, Cell Signaling Technology, Danvers, MA, USA) for 8 h at 4 °C. After further incubating with secondary antibodies (anti-rabbit IgG; 1:5000; catalog number. 7074S, Cell Signaling Technology, Danvers, MA, USA) for 120 min at 25 °C, the membrane was washed a final time. All protein bands were developed using enhanced chemiluminescence and visualized with a Tanon-5200 imaging system (Shanghai, China). 

### 2.7. Statistical Analysis

Statistical analysis was performed using the independent sample t-test or Pearson correlation analysis by using SPSS 20.0 statistical software (SPSS, Chicago, IL, USA). Results were presented as means ± standard deviation (S.D.). A *p*-value less than 0.05 (*p* < 0.05) was considered as significant.

## 3. Results

### 3.1. The Blood Glucose Changes of ApoE-/- Mice with STZ-Induced T2D

**Figure 1:**
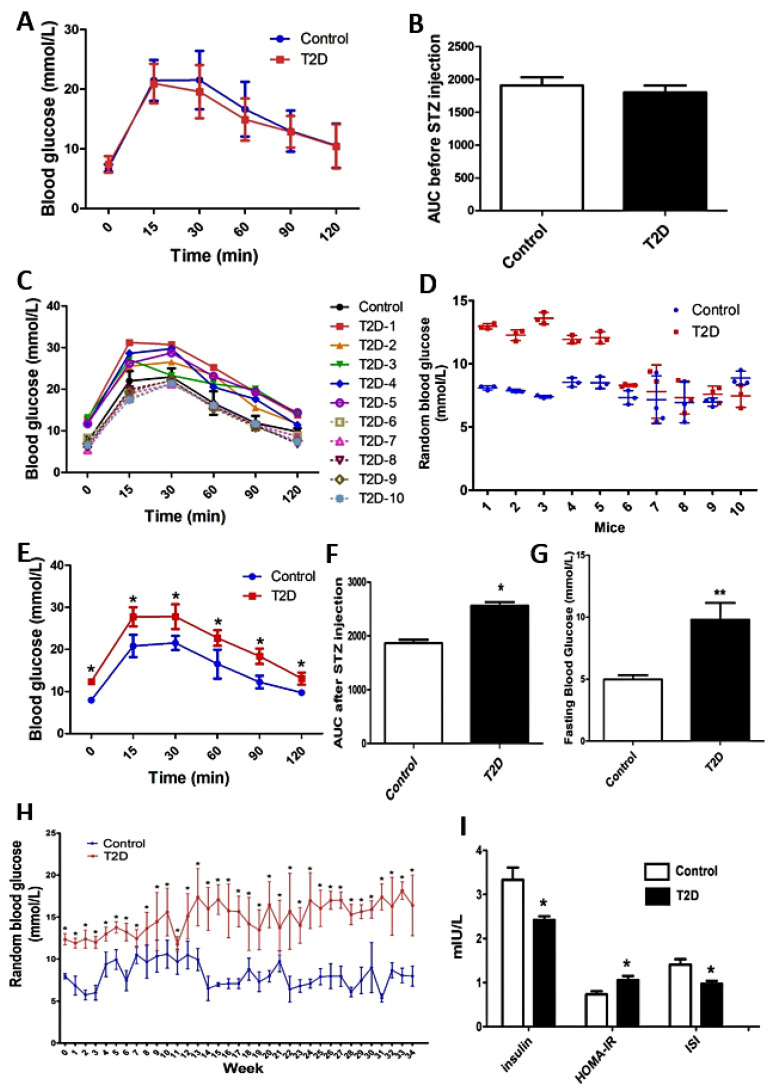
The induction of type 2 diabetes (T2D) and blood glucose changes in ApoE-/- mice. (**A**) An intraperitoneal glucose tolerance test (IPGTT) was performed on mice of each group (*n =* 10) before the injection of streptozocin (STZ) or vehicle, and (**B**) the area under the curve (AUC) was correspondingly calculated. (**C**) IPGTT was performed again on mice of each group (*n =* 10) after STZ injection. The IPGTT curve was plotted using the average blood glucose value of all mice in the control group (*n =* 10), but using the individual blood glucose value of each mouse in the T2D group (*n =* 10). (**D**) The random blood glucose levels of mice (*n =* 10) in each group were determined once a day for three consecutive days after STZ injection. The (**E**) IPGTT curve and (**F**) AUC value of mice in the final control (*n =* 5) and T2D (*n =* 5) group were presented after STZ injection. (**G**) The fasting blood glucose (FBG) of mice (*n =* 5) in each group was determined at the end of the experiment. (**H**) The random blood glucose (*n =* 5) was detected once a week during the whole period of the experiment. (**I**) The serum insulin levels of mice (*n =* 5) were determined using ELISA kits at the end of the experiment and the homeostasis model assessment for insulin resistance (HOMA-IR) and insulin sensitivity index (ISI) were correspondingly calculated. Data were statistically analyzed using the independent sample t-test in the SPSS 20.0 software. Results represent means ± S.D. of the samples (*n =* 10 or *n =* 5). *: *p* < 0.05 vs. control. **: *p* < 0.01 vs. control.

The HFD-fed ApoE-/- mice are a well-established model for studying atherosclerosis. T2D usually accelerates the progression of atherosclerosis. In this study, we used STZ to induce T2D in the HFD-fed ApoE-/- mice in order to investigate the progression of atherosclerosis in T2D mice. Before STZ injection, the AUC of IPGTT was not significantly different (*p* > 0.05) between mice in the control and T2D groups indicating no difference in their glucose metabolism (Figure 1A,B). Following STZ injection for three consecutive days, five out of the ten mice in the T2D group had a significantly higher (*p* < 0.05) AUC of IPGTT than mice in the control group (Figure 1C,E,F) and had random blood glucose levels ≥ 11.1 mmol/L (Figure 1D). Therefore, these five mice were judged as T2D mice and were finally included in the T2D group.

During the whole period (34 w) of the experiment, mice in the T2D group had significantly higher (*p* < 0.05) random blood glucose levels at all time points than mice in the control group (Figure 1H). At the end of the experiment, mice in the T2D group had significantly elevated (*p* < 0.05) fasting blood glucose levels (Figure 1G) and decreased (*p* < 0.05) fasting insulin levels (Figure 1I) than mice in the control group. Meanwhile, mice in the T2D group had a significantly higher (*p* < 0.05) HOMA-IR and a lower (*p* < 0.05) ISI compared to mice in the control group.

### 3.2. Animal Growth and Feed Consumption

**Figure 2:**
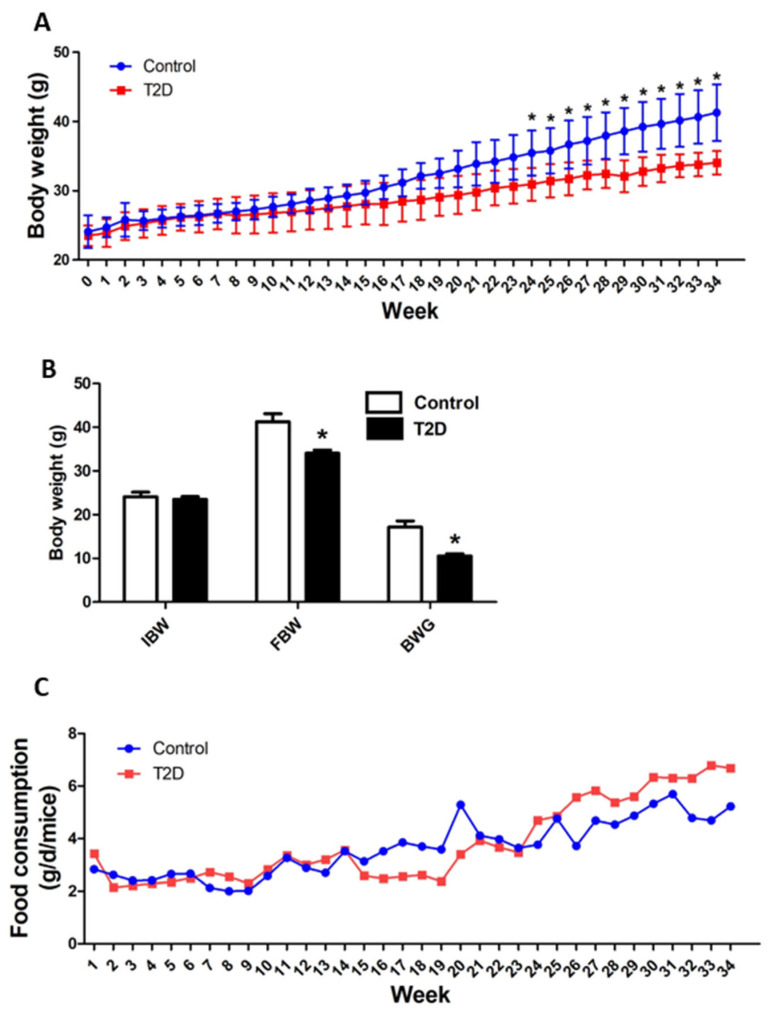
Animal growth and feed consumption. The body weight (BW) and feed intake of mice were monitored once a week. (**A**) The growth curve was plotted. (**B**) The initial body weight (IBW), final body weight (FBW), and body weight gain (BWG) were calculated. (**C**) The feed consumption throughout the experimental period was calculated. Data were statistically analyzed using the independent sample t-test in the SPSS 20.0 software. The result of feed consumption was not statistically analyzed because the five mice in each group were housed in one cage and the feed intake was recorded per cage. Results are presented as means ± S.D. of the samples from five (*n =* 5) mice in each group. *: *p* < 0.05 vs. control.

As seen in Figure 2A, the growth curve of mice increased gradually throughout the whole period of the experiment. Compared to the control mice, although the initial body weight (IBW) was not statistically different (Figure 2B), the STZ-induced T2D mice showed a slower growth speed with a statistically significant lower (*p* < 0.05) BW from week 24 to 34. Therefore, the STZ-induced T2D mice had a significantly lower (*p* < 0.05) final body weight (FBW) and body weight gain (BWG) compared to the control mice (Figure 2B). The feed consumption had no statistical analysis because the five mice in each group were housed in one cage and the food intake was recorded per cage (Figure 2C). Even so, it seems that the STZ-induced T2D mice consumed more feed than the control mice from weeks 24 to 34. These results indicated that the STZ-induced T2D mice had slower growth and reduced BWG compared with control mice.

### 3.3. ApoE-/- Mice with STZ-Induced T2D Had Accelerated Progression of Atherosclerosis

**Figure 3:**
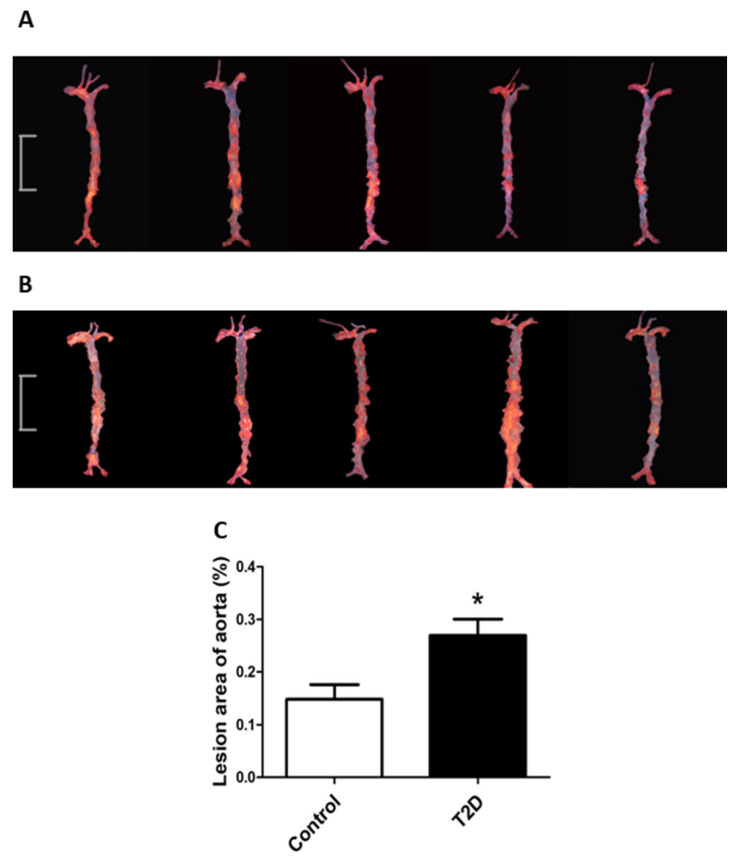
The progression of atherosclerotic plaques in the aorta was accelerated in streptozocin (STZ)-induced type 2 diabetic ApoE-/- mice. The aortas of the control (**A**) and T2D (**B**) mice were dissected and stained with oil red O. Scale bar is 1 cm (**A**,**B**). (**C**) The atherosclerotic lesions in the aorta were quantified using methods described in the Materials and Methods section. Data were statistically analyzed using the independent sample t-test in the SPSS 20.0 software. Results represent means ± S.D. of the samples from five (*n =* 5) mice in each group. *: *p* < 0.05 vs. control.

Obvious atherosclerotic plaques often develop in the large arteries of ApoE-/-mice fed with an HFD. In this study, we analyzed the atherosclerotic plaques in the aorta and quantified the atherosclerotic lesions. As shown in Figure 3, the STZ-induced T2D mice had significantly more severe (*p* < 0.05) atherosclerotic lesions in the aorta compared to the control mice. These results suggest that the progression of atherosclerosis was accelerated in the ApoE-/- mice with STZ-induced T2D.

### 3.4. ApoE-/- Mice with STZ-Induced T2D Had Decreased Cholesterol Synthesis but Increased Efflux in Liver

**Figure 4:**
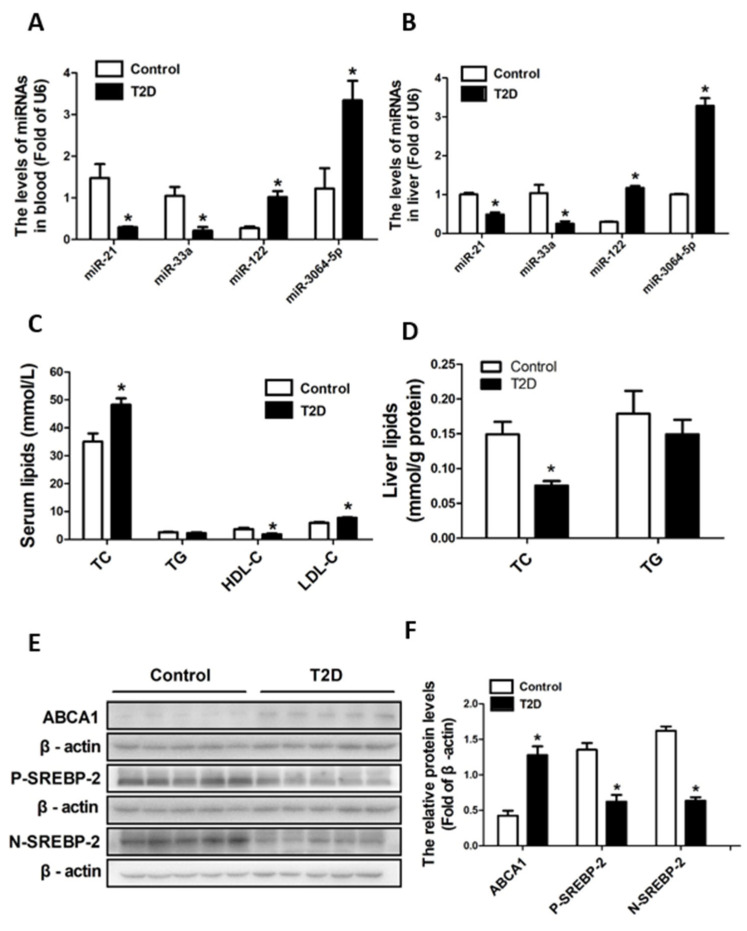
The miRNAs expression and lipid profiles in the blood and liver as well as the expression of key molecules for the synthesis and efflux of cholesterol in the liver. The relative expressions of miR-21, miR-33a, miR-122, and miR-3064-5p in the blood (**A**) and liver (**B**) were detected by using real-time fluorescent quantitative PCR. The levels of TC, TG, HDL-C, and LDL-C in the serum (**C**), and TC and TG in the liver (**D**) were determined by using commercial kits. The protein expressions of Abca1 and Srebp-2 in liver tissues were determined by western blotting (**E**,**F**). Data were statistically analyzed using the independent sample t-test in the SPSS 20.0 software. Results represent means ± S.D. of the samples from five (*n =* 5) mice in each group. *: *p* < 0.05 vs. control.

We measured the lipid profiles (TC, TG, LDL-C, and HDL-C) in both serum and liver tissues since it is well acknowledged that an HFD-induced disorder of lipid metabolism pivotally contributes to the progression of atherosclerosis. The ApoE-/- mice with STZ-induced T2D had significantly increased levels (*p* < 0.05) of TC and LDL-C and decreased levels (*p* < 0.05) of HDL-C in the serum (Figure 4C) compared to the control mice. Meanwhile, the ApoE-/- mice with STZ-induced T2D had significantly decreased levels (*p* < 0.05) of TC in the liver (Figure 4D). In addition, the levels of TG in the serum and liver were not significantly different (*p* > 0.05) between the T2D mice and the control mice (Figure 4C,D). These results suggest that the STZ-induced T2D ApoE-/- mice had increased TC and LDL-C levels in the blood but decreased cholesterol levels in the liver.

Cholesterol homeostasis is to a large extent determined by the synthesis/uptake and efflux of cholesterol in the liver. Therefore, we further looked at the expression of key molecules that are responsible for regulating the synthesis/uptake and efflux of cholesterol. SREBPs are key transcriptional regulators of genes involved in cholesterol biosynthesis/uptake. As seen in Figure 4E,F, the protein expressive levels of precursor Srebp-2 (P- Srebp-2) and mature Srebp-2 (N- Srebp-2) were significantly reduced (*p* < 0.05) in the liver tissues of the STZ-induced T2D ApoE-/- mice compared to that of the control mice. Meanwhile, the protein expressive level of Abca1, which is an important regulator of HDL synthesis and responsible for reverse cholesterol transport, was significantly elevated (*p* < 0.05) in the liver tissues of the STZ-induced T2D ApoE-/- mice compared to that of the control mice. These results indicate that the cholesterol synthesis in the liver was decreased but its efflux was increased in the ApoE-/- mice with STZ-induced T2D.

### 3.5. ApoE-/- Mice with STZ-Induced T2D Had Decreased miR-33a and miR-21 and Increased miR-122 and miR-3064-5p in Both Blood and Liver

Accumulating evidence shows that multiple circulating miRNAs are dysregulated in glucose and lipid metabolic disorders as well as their associated diseases such as atherosclerosis, etc. In this study, we analyzed several miRNAs in the blood and liver that might be involved in atherosclerosis. The expressions of miR-21 and miR-33a in the blood (Figure 4A) and liver (Figure 4B) were significantly downregulated (*p* < 0.05) in ApoE-/- mice with STZ-induced T2D. However, the expressions of miR-122 and miR-3064-5p in the blood (Figure 4A) and liver (Figure 4B) were significantly upregulated (*p* < 0.05) in ApoE-/- mice with STZ-induced T2D.

Moreover, we analyzed the relationship between the circulating miRNA levels and aortic lesion areas using Pearson correlation analysis (Table 2). The aortic lesions were positively correlated with the circulating levels of miR-122 (*r* = 0.997, *p* = 0.001) and miR-3064-5p (*r* = 0.951, *p* = 0.004), and negatively correlated with the circulating levels of miR-21 (*r* = −0.857, *p* = 0.029) and miR-33a (*r* = −0.912, *p* = 0.011) among all mice. In addition, the aortic lesions showed a positive correlation with miR-122 (*r* = 1.000, *p* = 0.001) and a negative correlation with miR-21 (*r* = −1.000, *p* = 0.001) in ApoE-/- mice with T2D.

Together, these results suggested that the aortic lesions correlate with the decreased miR-33a and miR-21 and increased miR-122 and miR-3064-5p in circulation of ApoE-/- mice with STZ-induced T2D.

## 4. Discussion

Many studies showed that the progression of atherosclerosis is accelerated in the case of T2D [2,7,18]. In this study, ApoE-/- mice fed with an HFD developed distinct atherosclerotic lesions in the aorta suggesting that it is a good and reproducible model for studying atherosclerosis. STZ injection caused hyperglycemia and insulin resistance in the ApoE-/- mice indicating the successful induction of T2D. In ApoE-/- mice with STZ-induced T2D, the development of atherosclerotic plaques was aggravated, which concurs with previous reports [2,7]. The mechanisms underlying the T2D-associated acceleration of atherosclerosis are still not completely understood although several mechanisms have been proposed [2]. MiRNAs have shown great potential for diagnostic or prognostic tests in diseases including atherosclerosis. The differential miRNAs expression patterns between common atherosclerosis and atherosclerosis combined with T2D may serve as biomarkers for screening cardiovascular risks in T2D. Here, the data indicated that several miRNAs (miR-21, miR-33a, miR-122, and miR-3064-5p) were potentially associated with the development of atherosclerosis in T2D.

Atherosclerosis is primarily characterized by lipid metabolism disorders and inflammation [19]. Several studies have investigated the role of miR-21, the most abundant miRNA in macrophages, in inflammation and lipid metabolism as well as in atherosclerosis. It is well-established that miR-21 played a key role in the inhibition of proinflammatory responses and the resolution of inflammation [20]. The absence of miR-21 results in accelerated atherosclerosis, plaque necrosis and vascular inflammation [21]. Therefore, miR-21 downregulation in circulation may favor vascular inflammation which creates suitable conditions for the formation of atherosclerotic plaques. Moreover, miR-21 reduced intracellular lipid accumulation induced by stearic acid in Hepa 1-6 cells by downregulating fatty acid-binding protein 7 (FABP7), a direct target of miR-21 [22]. Raitoharju et al. reported that the expression of miR-21 was significantly upregulated (fold changes 4.61) in atherosclerotic arteries compared to nonatherosclerotic left internal thoracic arteries [23]. Cengiz et al. found that the level of miR-21 in plasma was elevated in subclinical atherosclerosis in hypertensive patients compared to healthy controls [24]. However, Canfrán-Duque et al. reported that miR-21 depletion in macrophages triggered atherosclerosis plaque progression [25]. A more recent study from Telkoparan-Akillilar et al. found that the miR-21 level diminished 3.5 times in the blood samples of patients with atherosclerosis compared to healthy controls [26]. Results from animal models also observed downregulated miR-21 levels in microdissected fibrous caps of ruptured plaques [20]. Our present results indicated that miR-21 level in the blood and liver was downregulated in ApoE-/- mice with T2D. In STZ-induced T2D rats, miR-21 antagomir could improve insulin resistance and lipid metabolism disorder by upregulating the expression level of metalloproteinases 3 (Timp3) [27]. In diabetes-associated vascular dysfunction, miR-21 stimulated the proliferation of vascular smooth muscle cells (VSMC) by targeting specificity protein-1 (SPI) [28] and protected endothelial cells against high glucose-induced endothelial cytotoxicity probably by inhibiting the expression of the death domain-associated protein (DAXX) [29]. Although most studies supported that miR-21 has a negative association with lipid accumulation and inflammation and miR-21 downregulation favors the progression of atherosclerosis, there are still controversies regarding the role of miR-21 in atherosclerosis as well as in T2D-associated atherosclerosis. This could be due to the different populations, animal models, or sample sources used by different scientists resulting in the inconsistent observations of miR-21 levels. Furthermore, detailed investigations are awaiting to clarify the role of circulating miR-21 as a biomarker for atherosclerosis as well as T2D-accelerated atherosclerosis.

MiR-122 is one of the most abundant miRNAs expressed in the liver of both mice and humans [30]. As a liver-specific miRNA, miR-122 plays not only a central role in liver development, differentiation, and homeostasis but also a crucial role in hepatic cholesterol homeostasis and fatty acid metabolism. The antagonism of miR-122 has been found to diminish the expression of several genes associated with lipid metabolism (acetyl-CoA carboxylase alpha, ACC1; acetyl-CoA carboxylase beta, ACC2, etc.) and cholesterol synthesis (sterol regulatory element-binding protein 2, SREBP2, etc.) in the liver [31]. Esau et al. found that miR-122 inhibition decreased plasma cholesterol levels resulting in the improvement of steatosis in HFD-fed mice [32]. Willeit et al. indicated that circulating miR-122 levels were increased in subjects with metabolic syndrome or T2D suggesting it is a marker for disorders of hepatic lipid metabolism [33]. In addition to cholesterol metabolism disorders, the pathophysiology of atherosclerosis is also characterized by chronic inflammation, oxidation, and apoptosis. The miR-122 antagonism represents a potential therapeutic approach for atherosclerosis since miR-122 promoted proinflammatory factors and oxidant injury in the liver and cardiovascular system [10]. The anti-apoptotic role of miR-122 inhibition has been evidenced by miR-122 inhibitor greatly suppressing the ox-LDL-induced apoptosis in human aortic endothelial cells. The expression of miR-122 was significantly elevated in aortic endothelial cells of HFD-fed ApoE-/- mice [34]. This agrees with our present results that the expression of miR-122 in circulation and the liver was also significantly increased in ApoE-/- mice with T2D compared to the control. MiR-122 elevation has been found to aggravate insulin resistance in hepatocytes by targeting insulin-like growth factor 1 receptor (Igf-1r), indicating that it has a possible contribution to the progression of atherosclerosis in T2D [35]. Moreover, Li et al. found that miR-122 primarily originated from circulation endothelial cells and monocytes, and its level was elevated in patients with acute myocardial infarction compared with patients with unstable angina [36]. Wang et al. reported that the circulating levels of miR-122 were significantly elevated in patients with severe coronary atherosclerosis. Moreover, the serum miR-122 levels were positively correlated with atherosclerotic severity [37]. A recent study investigated the relationship of several miRNAs including miR-122 with subclinical atherosclerosis in subjects with metabolic syndrome and found that miR-122 was positively associated with the cardio-ankle vascular index (CAVI) and correlated negatively with the aortic pulse wave velocity (AoPWV). In addition, age and triglycerides enhanced the prediction of the AoPWV by miR-122 [38]. Wu et al. reported that miR-122 was upregulated in the aortic intima and serum of ApoE-/- mice induced by an HFD, and miR-122 inhibition repressed the atherosclerotic plaque progression and vulnerable plaque formation in ApoE-/- mice [39]. Collectively, these studies support that miR-122 correlates positively with atherosclerosis and its severity and suggest that miR-122 is a good biomarker for predicting the development of atherosclerosis. However, there is one exception, where circulating miR-122 levels were significantly downregulated in coronary artery disease patients, according to a recent study by Mishra et al. [40].

Cholesterol homeostasis plays an important role in the progression of atherosclerosis. SREBPs are key transcription regulators of genes involved in cholesterol biosynthesis/uptake. The expression of Srebp-2 is insulin-dependent since it was observed to be decreased in insulin-deficient mice and increased with the increase in supplemental insulin dosage in rat liver cells [41]. In this study, STZ-induced T2D resulted in the decline of insulin in the serum of ApoE-/- mice. Thus, the hepatic levels of Srebp-2 (P- Srebp-2 and N- Srebp-2) were correspondingly decreased in ApoE-/- mice with T2D compared to the control mice. In addition, the elevation of miR-122 expression may contribute to the decrease in Srebp-2 because it has been shown that the high miR-122 expression could inhibit the Srebp-2 expression in the liver [42]. MiR-33a embeds within the introns of the Srebp2 gene and is co-transcribed when Srebp2 is activated. Therefore, the hepatic downregulated expression of Srebp-2 led to the decline of miR-33a as observed in the present study. MiR-33a has target genes involved in cholesterol export, such as Abca1, Abcg1 and Niemann-Pick C1 (Npc1) [43]. As a main miR-33a target gene, Abca1 is essential for the biogenesis of HDL in the liver and is responsible for the movement of free cholesterol out of the cell which is called reverse cholesterol transport (RCT). The miR-33a-deficient mice had significantly higher plasma HDL-C levels compared to wild-type C57BL/6 mice [44]. The inhibition of miR-33a increased hepatic Abca1 and circulating HDL by as much as 40% [45]. The anti-miR-33a therapy enhances RCT and regresses atherosclerosis in LDL-R knockout mice [44]. Therefore, miR-33a inhibition has been considered a therapeutic method for elevating HDL and exhibits potential in the prevention of atherosclerosis. In the present study, the levels of miR-33a in the blood and liver were both decreased in ApoE-/- mice with T2D compared to the control mice. Meanwhile, the increased hepatic Abca1 level along with the elevated serum levels of TC and LDL-C were observed in ApoE-/- mice with T2D. These results indicate that T2D inhibited the expression of miR-33a and increased the cholesterol efflux in the liver of ApoE-/- mice. Increased liver cholesterol efflux which led to the elevation of TC and LDL-C in circulation may contribute to the aggravation of atherosclerosis in T2D. The silencing and inhibition of miR-33a usually results in HDL elevation in circulation, according to existing studies [44,45]. Interestingly, the miR-33a in the liver and circulation was decreased while the HDL-C in circulation was also decreased in ApoE-/- mice with T2D. It has been shown that the HDL generated by miR-33a inhibition was functional, and the absolute levels of plasma HDL cannot truly reflect the functional HDL which is responsible for the removal of cholesterol from peripheral tissues into the feces for excretion. Anyway, this awaits further investigation regarding the changes of total HDL-C and its functional forms during the T2D-accelerated progression of atherosclerosis. Collectively, these results suggested that the downregulation of miR-33a promoted hepatic cholesterol efflux contributing to aggravated atherosclerosis in ApoE-/- mice with T2D.

MiR-3064-5p is the mature isoform of miR-3064 located in chromosome 17q23.3 [13]. It has been shown that miR-3064-5p inhibits cementoblast differentiation [16] and regulates osteogenesis [46]. Furthermore, miR-3064-5p plays roles in the regulation of cancers including hepatocellular carcinoma [47], gastric cancer [13], bladder cancer [48], and breast cancer [49]. The expressions of miR-3064-5p were increased in both the blood and liver of ApoE-/- mice with T2D compared to the control mice. According to our unpublished study, miR-3064-5p targets the IκBα gene and activates NF-κB signaling in palmitate-stimulated RAW264.7 macrophage cell lines, indicating its role in the regulation of inflammation. Thus, miR-3064-5p possibly participates in the regulation of inflammation during the T2D-accelerated progression of atherosclerosis, which needs to be further elaborated.

## 5. Conclusions

In conclusion, STZ-induced T2D accelerates the progression of atherosclerosis in ApoE-/- mice. T2D-accelerated atherosclerosis correlates with decreases of miR-21 and miR-33a and the elevation of miR-122 and miR-3064-5p in circulation and the liver. This study adds a novel understanding of the potential roles of these miRNAs as biomarkers for predicting atherosclerotic progression in T2D.

## Figures and Tables

**Table 1:** Primer sequences.

Gene	Direction	Primer Sequence (5′-3′)
miR-21	RT	GTCGTATCCAGTGCAGGGTCCGAGGTATTCGCACTGGATACGACTCAACA
	F	CGGCGGTTAGCTTATCAGACTGA
	R	CCAGTGCAGGGTCCGAGGTAT
miR-33a	RT	GTCGTATCCAGTGCAGGGTCCGAGGTATTCGCACTGGATACGACTGCTT
	F	ACACTCCAGCTGGGGTGCATTGTAGTT
	R	CTCAACTGGTGTCGTGGAGT
miR-122	RT	GTCGTATCCAGTGCAGGGTCCGAGGTATTCGCACTGGATACGACCAAAC
	F	GTGACAATGGTGGAATGTGG
	R	AAAGCAAACGATGCCAAGAC
miR-3064-5p	RT	GTCGTATCCAGTGCGTGTCGTGGAGTCGGCAATTGCACTGGATACGACTTTGCA
	F	GGGTCTGGCTGTTGTGGTG
	R	CAGTGCGTGTCGTGGAGT
U6	F	CTCGCTTCGGCAGCACA
	R	AACGCTTCACGAATTTGCGT

**Table 2:** The Pearson correlation between circulating miRNAs levels and aortic lesion areas in the ApoE-/- mice with streptozocin (STZ)-induced type 2 diabetes (T2D).

miRNAs	All Mice (*n =* 6)		Control (*n =* 3)		T2D (*n =* 3)	
	r	*p*	r	*p*	r	*p*
miR-21	−0.857	0.029	−0.970	0.157	−1.000	0.014
miR-33a	−0.912	0.011	−0.986	0.105	−0.993	0.073
miR-122	0.997	0.001	0.767	0.443	1.000	0.001
miR-3064-5p	0.951	0.004	0.983	0.118	0.991	0.087

The Pearson correlation analysis was performed. A *p*-value less than 0.05 (*p* < 0.05) indicates statistical significance.

## Data Availability

The datasets generated during the current study are not publicly available due to the research content being the content of a graduate thesis, but are available from the corresponding author on reasonable request.
